# Comparison of the biological characteristics of human mesenchymal stem cells derived from exfoliated deciduous teeth, bone marrow, gingival tissue, and umbilical cord

**DOI:** 10.3892/mmr.2022.12919

**Published:** 2022-12-19

**Authors:** Jing Li, Shi-Qing Xu, Yu-Ming Zhao, Shi Yu, Li-Hong Ge, Bao-Hua Xu

Mol Med Rep 18: 4969–4977, 2018; DOI: 10.3892/mmr.2018.9501

Subsequently to the publication of this paper, an interested reader drew to the authors’ attention that, in Fig. 3 on p. 4973, the data panels shown for the “Osteogenesis” row of data for the GMSC and BMSC experiments appeared to be overlapping, such the data may have been derived from the same original source. After having examined their original data, the authors have realized that the data panel selected for the GMSC “Osteogenesis” experiment was inadvertently chosen incorrectly.

The corrected version of Fig. 3 is shown below. Note that this error did not significantly affect the results or the conclusions reported in this paper, and all the authors agree to this Corrigendum. The authors are grateful to the editor of *Molecular Medicine Reports* for allowing them the opportunity to publish this corrigendum, and apologize to the readership for any inconvenience caused.

## Figures and Tables

**Figure 3.**
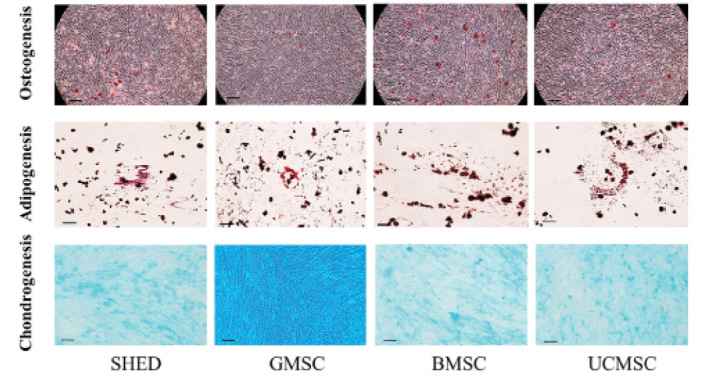
Osteogenic, adipogenic and chondrogenic differentiation of MSCs. MSCs were induced to differentiate toward osteogenic, adipogenic and chondrogenic lineages as verified by Alizarin Red, Oil Red O and Alcian Blue staining, respectively (osteogenic and chondrogenic differentiation: scale bar, 100 µm; adipogenic differentiation: scale bar, 50 µm). MSCs, mesenchymal stem cells; SHEDs, stem cells from human exfoliated deciduous teeth; UCMSCs, umbilical cord-derived mesenchymal stem cells; GMSCs, gingiva-derived mesenchymal stem cells; BMSCs, bone marrow mesenchymal stem cells.

